# Human Aquaporin-4 and Molecular Modeling: Historical Perspective and View to the Future

**DOI:** 10.3390/ijms17071119

**Published:** 2016-07-13

**Authors:** Giuseppe Felice Mangiatordi, Domenico Alberga, Daniela Trisciuzzi, Gianluca Lattanzi, Orazio Nicolotti

**Affiliations:** 1 Dipartimento di Farmacia-Scienze del Farmaco, Via Orabona, 4, University of Bari ”Aldo Moro”, 70126 Bari, Italy; giuseppe.mangiatordi@uniba.it (G.F.M.); daniela.trisciuzzi@uniba.it (D.T.); 2 Institut de Recherche de Chimie Paris CNRS Chimie ParisTech, PSL Research University, 11 rue P. et M. Curie, F-75005 Paris, France; domenico.alberga@chimie-paristech.fr; 3 INFN-Sez. di Bari and Dipartimento di Medicina Clinica e Sperimentale, University of Foggia, Viale Pinto, 71122 Foggia, Italy

**Keywords:** human aquaporin-4 (hAQP4), molecular dynamic (MD) simulations, druggability, gating mechanisms, epitope, neuromyelitis optica (NMO)-IgG binding

## Abstract

Among the different aquaporins (AQPs), human aquaporin-4 (hAQP4) has attracted the greatest interest in recent years as a new promising therapeutic target. Such a membrane protein is, in fact, involved in a multiple sclerosis-like immunopathology called Neuromyelitis Optica (NMO) and in several disorders resulting from imbalanced water homeostasis such as deafness and cerebral edema. The gap of knowledge in its functioning and dynamics at the atomistic level of detail has hindered the development of rational strategies for designing hAQP4 modulators. The application, lately, of molecular modeling has proved able to fill this gap providing a breeding ground to rationally address compounds targeting hAQP4. In this review, we give an overview of the important advances obtained in this field through the application of Molecular Dynamics (MD) and other complementary modeling techniques. The case studies presented herein are discussed with the aim of providing important clues for computational chemists and biophysicists interested in this field and looking for new challenges.

## 1. Introduction

Aquaporins (AQPs) are transmembrane channel proteins enabling the selective transport of water and other small solutes (such as glycerol) across cells [1,2,3,4,5,6]. AQPs can be divided into two different subfamilies: (1) AQPs selective only for water (corresponding in humans to AQP1, AQP2, AQP4, AQP5 and AQP8); (2) AQPs allowing the passage also of glycerol and other uncharged molecules (AQP3, AQP7, AQP9 and AQP10). The latter are also named “aqua-glyceroporins” [7]. AQPs are structured as tetramers, whose monomers constitute functionally independent pores. The AQP transport is extremely fast (about three billion water molecules per second [8]) and is operated in response to an osmotic pressure gradient occurring between the two sides of the membrane. AQPs play key biological roles, regulating the volume and internal osmotic pressure of the cells [9,10]. AQP dysfunctioning is behind the onset of several pathologies, associated to imbalanced water homeostasis [9,11,12] or autoimmunity [13]. Nowadays, the relevance of AQPs as potential pharmacological targets for therapeutics implications is widely acknowledged. In this respect, a breeding ground for cutting-edge research is the in-depth understanding of the water transport mechanism: this knowledge is essential to set up rational molecular strategies to address the design of selective ligands biasing AQPs [14]. Unfortunately, this goal is still largely unmet being the available experimental techniques such as X-ray crystallography or NMR spectroscopy insensitive to the very short timescales (in the order of nanoseconds) of water conduction events [8]. In this scenario, an unprecedented role has been played by molecular dynamics (MD), which is the front-line method for simulating at the molecular level the behavior of bio-macromolecules. In the present survey, we give an overview of the most recent applications of MD studies and complementary modeling techniques to study AQPs and provide valuable molecular information for the rational design of AQPs modulators. Importantly, the group of de Groot has shed light on crucial aspects of aquaporin biology by using MD simulations [7,15,16,17,18,19,20]. Herein, the focus is mainly on the understanding of the molecular mechanisms underlying functioning and modulation of human AQP4 (hAQP4), which is the most abundant water channel in the brain. An informed view of different case studies is given by emphasizing how the appropriate application of computational techniques can help in dissecting the complexity of hAQP4 physiology with the aim of providing a solid scientific platform for future drug design studies.

## 2. Human Aquaporin-4 (hAQP4), the Predominant Water Channel in the Central Nervous System

**Figure 1:**
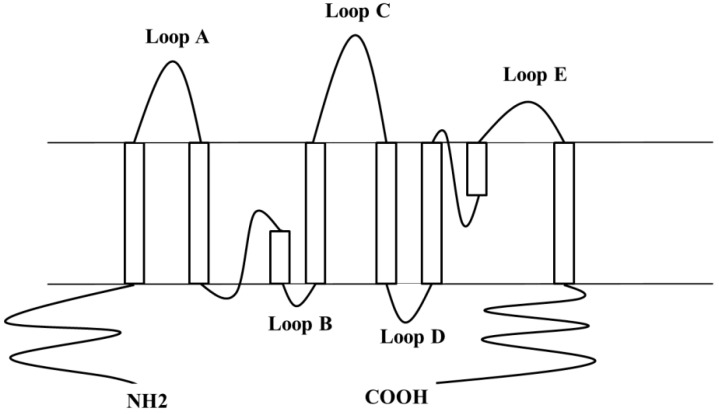
2D sketch of human aquaporin-4 (hAQP4) structural elements. hAQP4 is made of eight membrane-embedded helical segments, three extracellular loops (**loop A**, **loop C** and **loop E**) and two intracellular loops (**loop B** and **loop D**). Adapted from reference [22].

Like other AQPs, hAQP4 is a membrane protein characterized by six transmembrane helices forming a water-selective pore [21] (Figure 1).

Depending on the presence of 22 amino acids at the cytoplasmic N-terminus, two different hAQP4 isoforms have been identified, namely M1 and M23 [23,24]. As far as the localization in the human body is concerned, hAQP4 is expressed in the astrocytes and in different tissues that are in close contact with the blood vessels or with the cerebrospinal fluid [25,26]. Among others, hAQP4 has claimed great interest in recent years as a new promising therapeutic target. hAQP4 is involved in several disorders of water homeostasis (i.e., deafness [27,28], formation of brain edema [29,30], epilepsy [31], and lupus cerebritis [32]). More recently, hAQP4 has been found as the target antigen of IgG autoantibodies in a multiple sclerosis-like disorder named Neuromyelitis Optica (NMO) [13], a neuro-inflammatory demyelinating autoimmune disease that primarily affects the optic nerves and the spinal cord [33]. This finding paves the road to new therapeutic strategies to tackle NMO pathogenesis, for instance, by rationally designing new drugs able to interfere with NMO-IgG binding. Despite these important therapeutic implications, only few AQP4 modulators (see Table 1) have been developed so far [34,35,36,37,38], whose selectivity is questionable. Nevertheless, their discovery was made irrespective of a clear rationale based on the knowledge of AQP4 functioning and dynamics [39].

In this respect, molecular modeling is in the spotlight (a) to get insights into mechanism and kinetics of water conduction by unveiling the role of the key amino acid residues for the regulation of water permeation; (b) to identify the structural features of NMO-IgG binding; (c) to predict the druggability of this important albeit elusive target. Achieving these goals is an essential prerequisite for the rational design of small molecules acting as blockers of the hAQP4 function or as inhibitors of the NMO-IgG binding.

## 3. Why Molecular Modeling?

### 3.1. Understanding the Water Permeation Mechanism

Soon after the discovery of AQPs by iffuse through the H-bond network of water molecules (i.e., Grotthuss-based mechanism [40]). In thPeter Agre [41], experts sparked a passionate debate to elucidate the inexplicable molecular mechanism of the fast and highly selective water conduction of these channel proteins [42]. Despite the enormous efforts, for many years the scientific community was unable to clarify how such fast water transport could take place avoiding the conduction of protons [8], which are instead expected to de early 2000s, the release of the first high-resolution AQP structures allowed to hypothesize that water molecules move in a single row through the channel and that the lack of a continuous hydrogen bond network prevents proton conduction via a Grotthuss mechanism [3]. In this respect, MD studies [43,44,45] suggested a different mechanism of proton exclusion: protons cannot cross the channel due to the presence of a large electrostatic barrier originated by two alpha helices, namely HB and HE, and not as a consequence of an interrupted proton wire in the pore. In this picture, a crucial role is played by two NPA (Asparagine–Proline–Alanine) motifs, almost totally conserved among different AQPs [42], which work as a switch, reorienting each single water molecule as soon as it passes through the center of the pore. Several computational studies, explicitly considering the excess protons in the channels, supported this hypothesis. Meaningful examples are given by umbrella sampling MD simulations with the PM6 dissociable water model [46] and empirical valence bond proton transfer simulations [5,16,47]. Despite these efforts, the mechanism of proton exclusion in AQPs is far from being fully elucidated and is still a matter of debate [48]. In this regard, it is worth mentioning the recent paper by Urszula Kosinska Eriksson et al. [49]. The authors analyzed a sub-angstrom resolution X-ray structure and performed MD simulations of a yeast aquaporin, namely Aqy1. The authors not only detailed the occurring pairwise transport of water molecules but also showed that in addition to the electrostatic barrier originated by HB and HE, the particular orientation of water molecules at the SF region is also responsible for proton exclusion by preventing proton transport via a Grotthuss mechanism. Importantly, such a conclusion is consistent with experimental evidence indicating that mutations at the SF region can alter proton exclusion [6,50].

#### 3.1.1. hAQP4: X-ray Data and First MD Simulations

**Figure 2:**
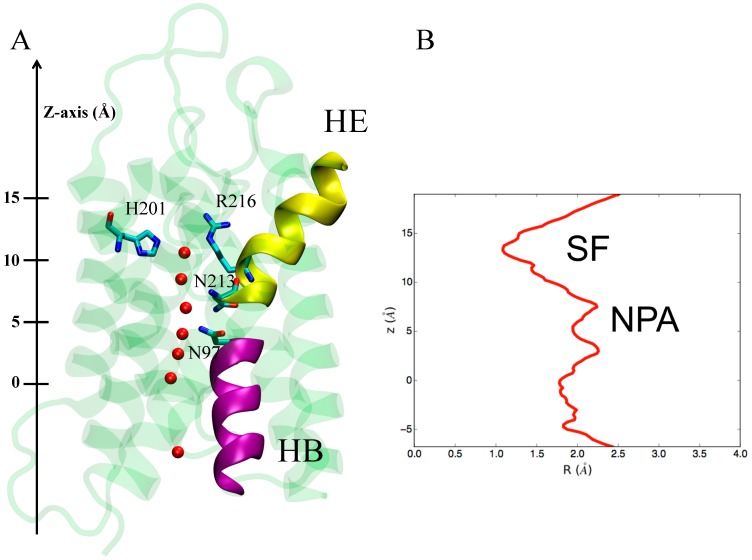
(**A**) X-ray structure of hAQP4. The X-ray solved structure of hAQP4 (PDB code 3GD8 [21]) is depicted as green cartoon representation. Important residues in the constricted selectivity filter (H201 and R216) and the asparagine residues belonging to the NPA motif regions are rendered as sticks. Water molecules inside the pore are shown as red spheres. The two short pore alpha helices responsible for an electrostatic barrier preventing proton conduction and named HE and HB are depicted as yellow and magenta cartoon representation respectively; (**B**) Pore radius. Pore radius R(Å) profile along the z-axis obtained from the hAQP4 X-ray structure using HOLE as cavity detection software (Department of Crystallography, Birkbeck Collage, University of London, London, UK) [51].

As far as hAQP4 is concerned, valuable insights into its mechanism of water conduction were obtained in 2009, with the milestone work of Joseph D. Ho et al. [21]. The authors reported for the first time the crystal structure of hAQP4. The X-ray solved structure shows a single file configuration of water molecules through the channel and the presence of a structural filter on the extracellular side of the channel, called a selectivity filter (SF), responsible for a pore constriction blocking the passage of solutes (Figure 2B), as reported for other water selective AQPs. In hAQP4, the SF consists of two residues, namely H201 and R216 (Figure 2A). Nevertheless, the X-ray data show an important difference with respect to other AQPs crystal structures: the asparagine residues belonging to the NPA motifs do not make a hydrogen bond with the same water molecule. This evidence suggests the possibility of a peculiar mechanism of conductance for this water channel with respect to other AQPs. MD simulations were performed to further investigate this issue. The ultimate aim was to assess the probability of finding a single central water molecule bonding to both NPA asparagine residues, as observed for other AQPs. Four different MD simulations were performed at different conditions: (1) X-ray coordinates of all protein atoms kept frozen and crystallographic water free to move (500 ps); (2) X-ray coordinates of all protein atoms kept frozen and crystallographic water molecules removed (500 ps); (3) heavy atoms of the protein restrained to their crystallographic positions by using harmonic restraints with free crystallographic water molecules (1 ns); (4) both protein and crystallographic water free to move (1 ns).

A change in terms of the configuration of water molecules inside the pore is observed if the protein is not kept fixed to its crystallographic position (simulations 3 and 4): the NPA asparagine residues established an H-bond with the same water molecule located in the middle of the pore, as occurs for other AQPs. In other words, the application of molecular modeling, and in particular of MD simulations, enabled the exclusion of a different mechanism of water conduction of hAQP4 with respect to other AQPs, despite a different configuration of water molecules in the X-ray crystallographic pose. The authors thus concluded that, similar to other water selective AQPs, the mechanism of conductance in hAQP4 can be summarized as follows: a single-row water permeation occurs through an amphipathic pathway where the side chains of F77, I81, V85, L170, I174 and V197 constitute the hydrophobic sites while the backbone carbonyl groups of G93, G94, H95 and I96 act as acceptors of hydrogen bonds from water molecules. Residues H201 and R216 form the SF while the NPA motifs (N97, P98, A99, N213, P214 and A215) are responsible for the already mentioned electrostatic barrier.

### 3.2. Computing Free-Energy Profile of Water Conduction

**Figure 3:**
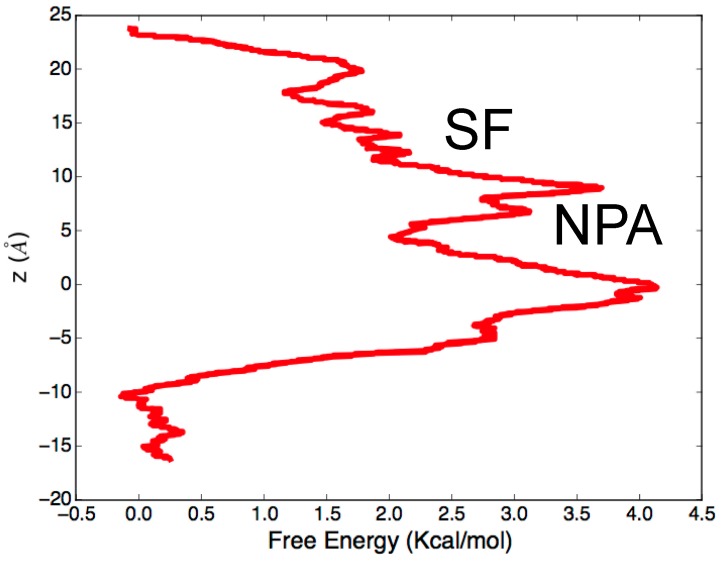
Free energy profile of water conduction in the hAQP4 water channel. Free energy landscape of the water transport in hAQP4 resulting from the application of Steered Molecular Dynamics (SMD) and Brownian dynamics fluctuation-dissipation theorems (BD-FDT). Data from ref. [52]. For the sake of clarity, z-axis values were scaled consistently with Figure 2.

Beyond describing the molecular mechanism of conductance, MD simulations can be also employed to estimate the free energy barrier associated with water permeation. In this respect, it is worth mentioning the paper by Cui and Bastien [52], published two years after the deposition of the first X-ray structure of hAQP4. The authors not only confirm, through equilibrium MD simulations performed on the hAQP4 tetramer embedded in a lipid bilayer, the already described mechanism of water conductance, but also performed steered molecular dynamics (SMD) [53], a computational technique that allows the improvement of sampling of standard MD simulations and to compute free energy barriers associated with the investigated process. The basic idea of SMD consists of the application of an external force to some atoms of the model system in order to observe a rare event, otherwise very difficult to sample within equilibrium MD. The free energy profile is finally obtained on the basis of the Brownian dynamics fluctuation-dissipation theorems (BD-FDT) [54] by which it is possible to extract equilibrium free-energy differences from non-equilibrium simulations, such as SMD. As far as hAQP4 is concerned, water permeation inside the pore, although very fast, takes place in response to an osmotic pressure gradient occurring at the interface of the membrane. Due to the applied boundary conditions, equilibrium MD of hAQP4 tetramer in a single lipid bilayer cannot reproduce such a pressure gradient. Instead, a larger model system, characterized by at least two membrane bilayers, should be implemented to actually separate cytoplasmic and extracellular compartments. Although this approach, which recently proved effective in computational electrophysiology [55], could be employed to study water transport in hAQP4, it requires very high computational resources. In other words, both the observation of the hAQP4 “at work” and the computation of the free energy associated with water permeation by equilibrium MD simulations are prohibitive from a computational point of view. In this regard, the work by Cui and Bastien [52] has indicated a viable strategy to get around such implicit MD limitation. Starting from the last frame of the trajectory resulting from 11 ns of equilibrium MD simulations, the authors pulled four water molecules in the system (one molecule in each monomer) and sampled, for each water molecule, ten pulling paths (five in each direction). By applying BD-FDT on the sampled paths, they obtained a complete free energy landscape of the water transport in hAQP4 (Figure 3). As expected, the most relevant free energy barrier (about 4 kcal/mol) was observed at the level of NPA motif responsible for a split of the pore into two different sub-pores. The passage from sub-pore to another involves a water reorientation requiring high values of energy to take place. As far as the SF is concerned, the energy cost for water permeation is much lower, thus suggesting that this domain, despite hindering the passage of solutes, has an almost negligible effect on the energy associated to water crossing.

### 3.3. Identifying Gating Sites

The simulations described so far gave insights into the hAQP4 water selectivity and the energy associated with the water conduction mechanism. However, these studies did not provide any information about water flux regulation. Experimental evidence suggests that gating mechanisms acting under the control of external stimuli, altering the cellular microenvironment, frequently regulate AQPs. In particular, changes in divalent cation concentrations [56,57,58], osmolality [59,60], and pH [56,57,61] as well as phosphorylation of single residues can affect water permeability of AQPs, including hAQP4 [62,63]. In this regard, noteworthy is the recent paper by Philip Kitchen et al. [64]. Starting from previous crystallographic analyses and in-silico predictions, the authors provided for the first time in vitro evidence whereby AQP4 water permeability is altered by site-directed mutations. More specifically, mutations at the SF region were taken into account. Building on this experimental evidence, it is possible to speculate that the molecular mechanism underlying the water flux regulation is likely related to conformational changes of specific protein portions or even single key residues (i.e., gating sites) controlling the opening/closure of the pore. A full understanding of these mechanisms can help to address the rational design of ligands that can act as modulators of water flux by engaging specific interactions with key protein residues. MD simulations proved effective to obtain valuable insights into gating sites of different AQPs [65,66,67].

#### 3.3.1. Identification of H95 as a New Gating Residue in hAQP4

**Figure 4:**
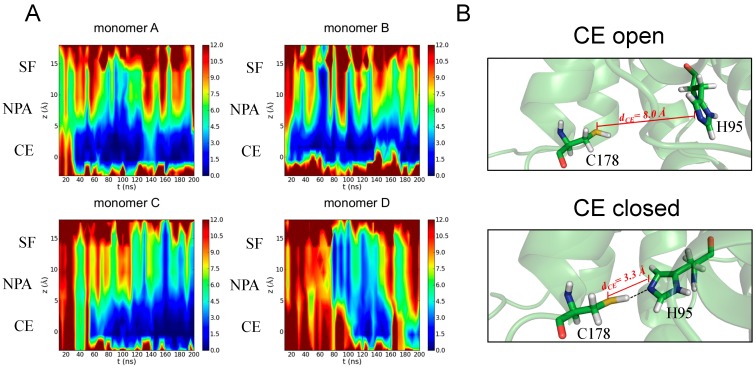
Gating site at the the cytoplasmic end (CE) region. (**A**) Color plots depicting the time dependence of the local osmotic permeability (10^−14^ cm^3^/s) computed for the four monomers; (**B**) Selected frames showing different states (**open** and **closed**) of the CE region. The H-bond interaction occurring between C178 and H95 residues and causing the closed state is depicted by a dotted line. Adapted from reference [22].

As far as hAQP4 is concerned, an early effort in this direction is represented by our recent study [22]. With respect to previous works, the extension of the time span of our analyzed MD trajectory (195 ns) allowed the observation for the first time of spontaneous fluctuations strongly reducing the total osmotic permeability and, consequently, identifying a gating site. In particular, we followed the approach by Masanori Hashido et al. [68] enabling the calculation of the osmotic permeability (Pf) based on equilibrium MD simulations performed on a hAQP4 tetramer embedded in a lipid bilayer. Indeed, valuable information about the channel dynamics can be obtained by connecting such a scalar quantity with the observed variation of the pore structure during the simulation. The time evolution of the local osmotic permeability along the z-axis of the channel, computed for each monomer, is shown in Figure 4A: all the monomers exhibit a sharp decrease of Pf during the simulation that can be ascribed to a partial closure of the pore occurring at different times and located at the cytoplasmic end (CE) of the channel. In order to provide a molecular rationale behind the p_f_ change observed during the simulation, we performed a closer inspection at the level of pore portions showing that water permeability decreases as a consequence of the rotation of the side chain of a histidine residue (H95) leading to a hydrogen bond interaction with a cysteine (C178), as reported in Figure 4B.

In other words, the analysis of MD simulations allowed us to complement the picture obtained from the already described previous studies: the residues forming the SF and NPA regions are responsible for water selectivity while one residue located at the cytoplasmic end of the channel, namely H95, seems to be crucial for water flux regulation. Importantly, the sequence alignment analysis [69] revealed that this residue is conserved within plant, humans, rats, and mice and is, thus, a key structural element for AQP4 functioning. Furthermore, the histidine is the only amino acid whose side chain has a pK_a_ of approximately 6.0, a value highly sensitive to the physiological pH. Even subtle pH variations occurring in specific biological microenvironments can in fact cause a remarkable modification of its protonation state. Indeed, a role of histidine residues in pH-mediated mechanisms has been suggested for many other proteins [70,71,72]. As a result, it could be postulated that H95 residue operates through a pH-based gating mechanism in hAQP4. Inspired by this hypothesis, Shreyas Kaptan et al. [73] recently demonstrated that “AQP4 can be regulated by intracellular pH through a change in protonation of H95”. Such important conclusions of the results come from a combined experimental/theoretical approach mixing MD simulations with in vitro studies. The authors performed equilibrium MD simulations (500 ns) on two different systems, one having H95 in a neutral state, the other having H95 in a protonated (positive charged) state. The obtained trajectories were analyzed by means of partial least squares based functional mode analysis (PLS-FMA) [74]. Such a methodology allows the identification of specific collective motions of the protein that can be causatively associated to the function of interest, in this case the opening of the pore resulting from the H95 protonation. The authors identified one correlated mode consisting of a side chain reorientation of H95 allowing an ionic interaction with E41, responsible for the pore opening. Such interaction takes place only if H95 is positively charged, thus, suggesting a pH-mediated gating mechanism at the cytoplasmic side of the channel. To experimentally test this hypothesis, the authors measured hAQP4 osmotic permeability: (1) at physiological pH; (2) after acidification of the extracellular compartment; (3) after acidification of the intracellular compartment. No changes were observed between the first and the second measures while the acidification of the intracellular compartment led to an increase of water permeability. Importantly, such an effect cannot be observed if the same measures are carried out on a mutant form of hAQP4, having an alanine in place of an histidine at position 95, thus supporting the hypothesis, suggested by MD simulations, whereby H95 might by responsible for the observed pH-mediated gating mechanism. Considered together, our recent work and the subsequent study of Shreyas Kaptan et al. [73], represent an unprecedented and successful example of how experiments can be rationally addressed when assisted by validated computational strategies.

### 3.4. Rationalizing Pathological Mutations

**Figure 5:**
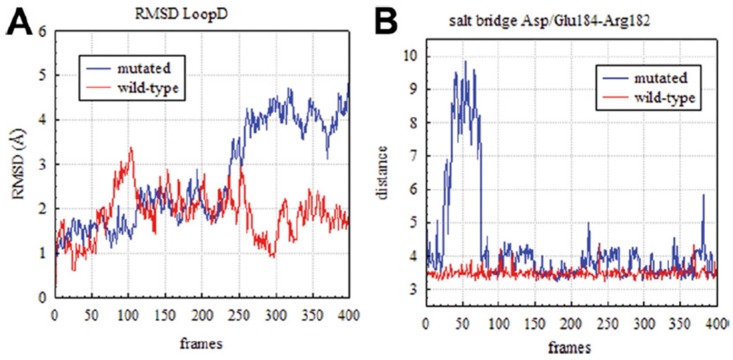
Effect of D^184^E mutation. (**A**) Root-mean-square deviations (RMSD) computed with respect to the 3GD8 crystal structure [21] for the C-alpha atoms belonging to the hAQP4 loop D for both mutated (blue line) and wild-type (red line) form; (**B**) Time-dependence of the distance between the center of mass of the oxygen atoms in the acidic side chain of residue at position 184 and the center of mass of the nitrogen atoms in the side chain of R182, adapted from reference [27]. Coordinates were saved every 3 ps (400 frames for a total simulation time equal to 1.2 ns).

It is widely acknowledged that structural dynamics and function of proteins are closely related [75]. As a result, even a single residue mutation in a given protein sequence can alter such structural dynamics thus affecting its function and, in some cases, causing a disease [76]. In this respect, comparative molecular modeling techniques can be used to assess the dynamics effects of some specific residue mutations with respect to the wild type (WT) form, whose structure is more often available from the protein data bank (PDB). These comparative studies are largely pursued to find a rationale for pathological polymorphisms mainly in those proteins, such as hAQP4, with a high therapeutic promise [77,78,79]. In particular, a mutational screening of the hAQP4 gene performed on different patients recently revealed the presence of single nucleotide polymorphism (SNP) on one subject exhibiting hearing loss [27]. Such SNP, corresponding to the substitution of an aspartate at position 184 with a glutamate (D^184^E mutation) strongly impacts hAQP4 water permeability [27]. The connection, at molecular level of detail, between such loss of functionality and the detected SNP may provide important clues about protein functioning and indicate putative rational strategies to contrast the effect of the considered pathological mutation. The molecular mechanism whereby the D^184^E mutation induces a loss of hAQP4 function was provided by MD simulations published in a co-authored work [27]: the replacement of a glutamate in place of an aspartate at position 184 resulted in a higher conformational mobility of the intracellular loop D. This increase in the conformational freedom forces loop D to obstruct the pore at the cytoplasmic side, hampering water crossing inside the channel. Such a conclusion was drawn by comparing the MD trajectories obtained from both the WT and the D^184^E forms, an approach already having been proved effective to shed light on the effect of point mutations on structural dynamics of several proteins [80,81,82,83]. The analysis revealed a different time-dependence of the root-mean-square deviations (RMSD) computed for the C-alpha atoms belonging to the loop D (see Figure 5A). Furthermore, it was clear from the simulations that such different behavior was likely due to the loss of the ionic interaction between the basic residue at position 182 (arginine) and the mutated residue at position 184 (see Figure 5B). This retrospective study provides a meaningful illustration of how molecular modeling can be effective in shedding light on the molecular mechanism behind known pathological mutations. Of course, molecular simulations can be also employed to address experimental studies when they are in progress. However, numerous examples of prospective applications have been reported in seminal papers describing how modeling can successfully guide site-directed mutagenesis experiments to identify key specific residues for protein function, thus being the mutation likely responsible for the onset of possible pathogenesis events [84,85,86,87,88].

### 3.5. Characterizing Epitope Structural Features

An increasing interest towards hAQP4, with respect to other AQPs, has been developed due to its involvement in an autoimmune inflammatory disorder of the central nervous system, called NMO. It is in fact well known that hAQP4 is recognized by NMO-specific serum autoantibodies (NMO-IgG) [13] and that the resulting interaction is responsible for complement-dependent cytotoxicity and antibody-dependent cell-mediated cytotoxicity [89,90,91]. Despite these widely accepted evidences, a specific treatment of NMO is still missing [39]. Current approaches are based on a generic suppression of the immune system with consequent severe side effects [39]. The delay in the development of new and more specific diagnostic and therapeutic tools is mostly due to the gap of knowledge of the NMO-IgG binding mechanism. In this respect, the most intriguing question is: which molecular events do regulate and characterize the molecular recognition of NMO-IgG to hAQP4? Several experimental works [92,93,94] pointed out that complex structural rearrangements (mainly involving the extracellular portion of AQP4) are required in order to assemble the hAQP4 epitope. In this respect, we very recently formulated the first hypothesis about the hAQP4 epitope molecular features through the combined application of mutagenesis experiments and MD simulations [95,96].

**Figure 6:**
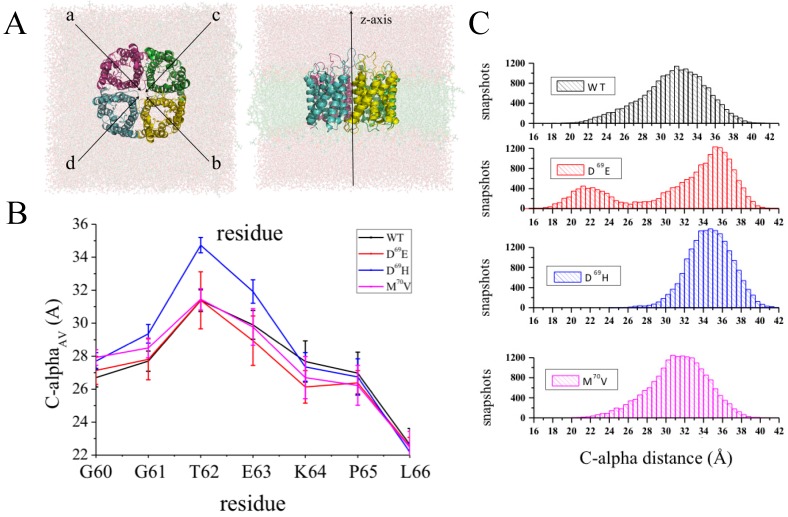
Conformational “domino effect” resulting from D69E and D69H mutations. (**A**) Top (**left**) and lateral (**right**) view of the investigated system; (**B**) C-alpha_AV_ values computed for residues belonging to a portion of the loop A (from residue G60 to residue L66); (**C**) Distance distribution function of C-alpha distances computed for residue T62. The uncertainties related to C-alpha distances are computed by the block averaging method [97]. Adapted after permission from reference [96].

As a first step, experiments showed that the substitution of an aspartate in position 69 (D69) with either a glutamate (D^69^E mutation) or a histidine (D^69^H mutation) strongly impairs binding between hAQP4 and the NMO-IgGs. It is worth noting that this residue is part of the second transmembrane region of the protein while it is acknowledged that NMO-IgG interacts with the easier accessible hAQP4 extracellular loops. In other words, this experimental observation cannot be explained by simply assuming that D69 is directly involved in NMO-IgG binding. In this respect, the effects of D69 should be investigated at a higher level of complexity in order to study the causative occurrence of specific molecular events determining the formation of a conformational epitope for NMO-IgG binding. On this premise, we carried out MD simulations of the WT hAQP4 tetramer and of different mutated forms, namely D^69^E, D^69^H, and M^70^V. The latter was taken into account as a negative control, being the interaction with NMO-IgG insensitive to M^70^V mutation. We aimed at exploring the putative conformational effects of the considered mutations on the extracellular loops by comparing the WT trajectory with those resulting from the simulated mutated forms. In particular, taking advantage of the system symmetry (axially-symmetric tetramer with respect to the z-axis, see Figure 6A), a new geometrical parameter was introduced and computed for each residue to account for its conformational behavior during the simulation, i.e., the distance between its C-alpha atom in a given monomer and that of the same residue in the specular monomer. For both occurrences (monomer A vs. monomer B and monomer C vs. monomer D), a value averaged along the trajectory was considered and the two resulting distances were further averaged in order to have a single value for each residue, namely C-alpha_AV_. Such analysis revealed that the substitution of a single residue in position 69 induces conformational changes of other residues upstream of the mutation involving the extracellular loop A (Figure 6B). Such a conformational “domino effect” is evident in D^69^H and mainly effective on a threonine in position 62 (T62). A more in-depth analysis, taking into account the distance distribution of C-alpha for T62 (Figure 6C), revealed that this effect occurs also in D^69^E, the other mutated form impairing the binding with NMO-IgG. In summary, MD simulations allowed us to speculate that D69 is a key element for the remote control of the T62 conformation and is likely crucial for hAQP4 epitope reorganization.

From a mere computational point of view, the present case study provides the opportunity to handle one of the most overlooked issues related to biomolecular simulations: the quality of molecular sampling. Ideally, several simulations should be performed before drawing conclusions from MD data. However, this is often unfeasible when the system under investigation is very large. In this context, simulating a tetramer is inherently of help: assuming that each monomer behaves independently, a single simulation can be considered as four independent MD simulations of hAQP4 monomers. In this respect, the robustness of the data can be confirmed by the evidence that the observed differences also occur when considering each monomer separately. Since the timescale for fluctuations is in the order of several nanoseconds, we applied the block averaging method [97], a recently developed statistic technique that is able to quantify the uncertainty and sampling quality of biomolecular simulations (the interested reader is referred to reference [97] for methodological details). In particular, this technique allows accurate estimates of the errors and of the time correlation associated to a given observable. We applied this methodology to the C-alpha distances computed for the extracellular loops. Importantly, a time correlation of about 5 ns was found, thus indicating a sufficiently high number of effective samples (19 for each monomer), the analyzed trajectories being 95 ns long. We do believe that it is wise to follow such modus operandi each time one aims at obtaining information from MD simulations of large biomolecules, as is the case with AQPs.

### 3.6. Assessing Druggability

**Figure 7:**
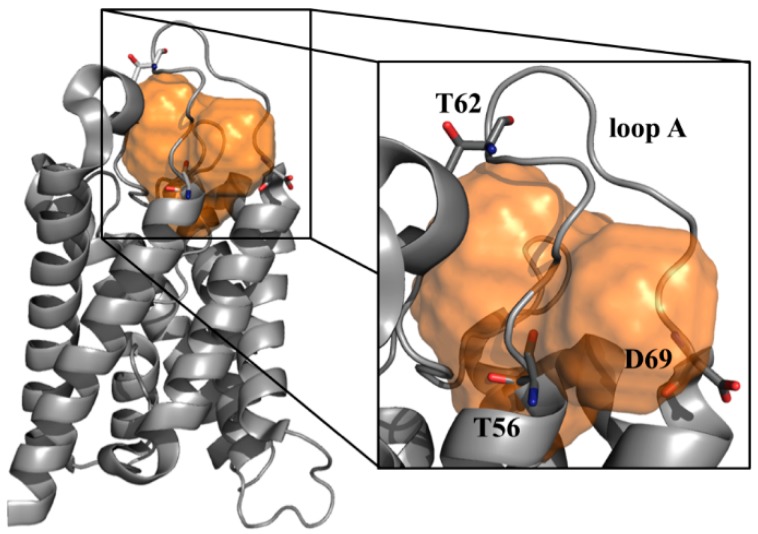
Identified druggable cavity in hAQP4. Cavity identified in the hAQP4 crystal structure (PDB entry: 3GD8 [21]) by the FLAPsite algorithm. As shown in the zoomed panel, the sole cavity found is located in proximity of the extracellular loop A and close to residues D69, T62 and T56. Adapted from reference [96].

All the case studies discussed so far aimed at providing breakthrough strategies to rationally address the design of small-molecules targeting hAQP4. However, the obtained important molecular clues would be worthless in the absence of a specific feature called “druggability” and defined as the ability of a given protein to “bind drug-like compounds with a binding affinity below 10 µM” [98]. Alan S. Verkman et al. in a recent review warned the scientific community about the fact that AQPs could be devoid of druggability, calling for greater attention for this important issue since “challenges associated with the development of better modulators include the druggability of the target” [99]. More generally, accurately predicting the druggability of a putative pharmacological target is highly desirable since it can strongly reduce the costs associated to the so-called “target selection” phase in the early drug discovery [100]. Different computational tools have been developed in the last few years to assess protein druggability [101,102,103,104,105] and one of them, named Volsurf [106], has been applied to hAQP4 [96] for the first time ever in our recent study [96]. In particular, all the potential cavities existing in the crystal structure of hAQP4 (PDB code 3GD8 [21]) were searched using an algorithm called FLAPsite [107,108]. It is based on a geometric approach which is able to find hydrophobic cavities starting from the 3D structure of a protein and employing GRID molecular interaction fields (MIFs) [109]. Figure 7 shows the sole cavity found by FLAPsite in hAQP4 potentially able to accommodate small molecules. Importantly, the druggability of such a site was assessed by computing several MIFs based descriptors and comparing the obtained values with those resulting from a reference dataset of cavities belonging to 43 X-ray solved pharmaceutically relevant targets [110]. This analysis unveils that all the computed physico-chemical properties are in the same range of the considered dataset thus suggesting that the found cavity is able to accommodate drug-like compounds and, therefore, can be considered for further rationally-based drug-design strategies.

## 4. Future Challenges

After a few years from the deposition in the PDB of its crystal structure [21], hAQP4 seems to be much less “mysterious” thanks to the compelling advances provided by molecular modeling. However, although progress in the field has been undoubtedly fast, several challenges must be addressed before all the collected evidence can be useful to develop hAQP4-targeting compounds. Such challenges involve several issues where molecular modeling can still play a central role. In this section we summarize these issues by highlighting relevant pieces of information for computational chemists and biophysicists interested in this field.

### 4.1. Investigating Orthogonal Array of Particles (OAPs) Aggregation

It is acknowledged that NMO-IgG recognizes hAQP4 only when organized in the plasma membranes in supra-molecular assemblies, called Orthogonal Array of Particles (OAPs) [94]. This experimental observation suggests that hAQP4 aggregation is required for the proper conformational rearrangement of the NMO-IgG epitopes and provides, for the first time, a rationale for the tissue-specificity of NMO associated damages. A different propensity of hAQP4 to aggregate has been, in fact, detected in different tissues [111]. In other words, the investigation of the OAPs aggregation process could be of utmost importance to understand NMO pathogenesis and shed light on the hAQP4 epitope aggregation. However, to the best of our knowledge, this investigation has never been carried out. This is likely due to the fact that the hAQP4 N-terminal portion, proved to be crucial for OAPs aggregation [112], has not been solved yet. Hence, these missing structural details have prevented the performance of reliable standard MD simulations. In this context, one of the most intriguing challenges of molecular modeling would be that of predicting the conformation, secondary structure (if present), and role in OAPs aggregation of such N-terminal protein portion. Until a few years ago, such a goal seemed unreachable. At present, specialized enhanced sampling techniques (e.g., Replica Exchange with Solute Tempering–REST2 [113]) and the ever growing availability of high performance computing resources could really provide breakthrough insights to understand NMO pathogenesis, thus making the simulation of OAPs aggregates feasible.

### 4.2. Structure-Based Virtual Screening

Virtual Screening (VS) is a computational approach widely employed by both academia and industry for a cost-effective lead discovery and optimization [114,115]. For the purposes of drug discovery, a typical VS procedure is carried out on large databases comprising thousands or even millions of drug-like molecules (i.e., commercial compounds) exploring a chemical universe as large as possible. The selected ligands can be then purchased and experimentally tested. To be successful, VS will have benefit of some preliminary information regarding the activities of known ligands (ligand-based VS [116]) or related to the 3D structure of the considered target (structure-based VS [117]). To the best of our knowledge, such an approach has never been applied to hAQP4. Actually, ligand-based VS studies are extremely challenging as only few hAQP4 selective compounds are known [34,35,36]. Anyhow, even structure-based VS procedures have never been employed, despite the fact that the X-ray solved structure has been available in the PDB since 2009 [21]. The main reason lies in the difficulty of mapping putative binding sites suitable as a potential target for drug-like compounds. Today, such limitation could be overcome since a cavity at the bottom of the loop A has been recently hypothesized as a potential binding site for drug like molecules [96]. We are certainly aware that the risk-reward ratio associated with a VS structure based strategy is still very high. Protein dynamics for drug design is still a very difficult task, although today different methodologies allow this issue to be approached. Examples are given by side-chain flexibility soft docking [118], induced fit [119], and conformational ensemble-based docking [120]. Moreover, no experimental evidence proving the druggability of the proposed cavity is yet available. Despite this, we do believe that the interest expressed by the scientific community in rationally developing hAQP4 modulators makes this approach a bet worth taking.

### 4.3. Drug-Repurposing Strategies

Another strategy recently proved to be effective for the identification of novel active compounds is the so-called drug repurposing [121]. It consists of using compounds with a known pharmacological action, previously developed for a diverse target, for new and often very different therapeutic purposes. Valuable examples are given by the use of thalidomide in severe erythema nodosum leprosum [122] as well as sildenafil in erectile dysfunction [123]. Obviously, repositioning existing drugs for new therapeutic uses allows bypassing the first steps typically required for the development of new drugs, going directly to preclinical testing and clinical trials, thus reducing associated risks and costs [124]. The identification of a druggable cavity makes the application of such strategy feasible also for hAQP4. In particular, cross-relationships can be searched between the found cavity and those of known targetable proteins by screening the entire dataset, as implemented in the FLAPSite algorithm. Ligands known to bind cavities very similar to that identified in hAQP4 can then be purchased and tested.

## Figures and Tables

**Table 1:** Compounds known to inhibit water permeability of AQP4 and to block Neuromyelitis Optica (NMO)-IgG binding.

Compound	Effect on AQP4	Reference
2-(Nicotinamido)-1,3,4-thiadiazole	Inhibition of water permeability	[36,37]
Sumatriptan	Inhibition of water permeability	[37]
Rizatriptan	Inhibition of water permeability	[37]
Acetazolamide	Inhibition of water permeability	[34]
Arbidol	Blockage of NMO-IgG binding	[38]
Berbamine	Blockage of NMO-IgG binding	[38]
Tamarixetin	Blockage of NMO-IgG binding	[38]

## Abbreviations

hAQP4	Human Aquaporin-4
NMO	Neuromyelitis optica
MD	Molecular Dynamics
SMD	Steered Molecular Dynamics
BD-FDT	Brownian dynamics fluctuation-dissipation theorems
VS	Virtual Screening
