# Erratum: Mangiatordi, G.F., et al. Human Aquaporin-4 and Molecular Modeling: Historical Perspective and View to the Future. *Int. J. Mol. Sci.* 2016, *17*, 1119

**DOI:** 10.3390/ijms17101720

**Published:** 2016-10-13

**Authors:** International Journal of Molecular Sciences Editorial Office

**Affiliations:** MDPI AG, St. Alban-Anlage 66, 4052 Basel, Switzerland; ijms@mdpi.com

The following changes have been made to the published paper [1]. The passage in Section 3.1 is as follows: “Soon after the discovery of AQPs by iffuse through the H-bond network of water molecules (i.e., Grotthuss-based mechanism [2]). In thPeter Agre [3], experts sparked a passionate debate to elucidate the inexplicable molecular mechanism of the fast and highly selective water conduction of these channel proteins [4]. Despite the enormous efforts, for many years the scientific community was unable to clarify how such fast water transport could take place avoiding the conduction of protons [5], which are instead expected to de early 2000s, the release of the first high-resolution AQP structures allowed to hypothesize that water molecules move in a single row through the channel and that the lack of a continuous hydrogen bond network prevents proton conduction via a Grotthuss mechanism [6]”. This passage should be replaced with the following: “Soon after the discovery of AQPs by Peter Agre [3], experts sparked a passionate debate to elucidate the inexplicable molecular mechanism of the fast and high selective water conduction of these channel proteins [4]. Despite the enormous efforts, for many years the scientific community was unable to clarify how such a fast water transport could take place avoiding the conduction of protons [5], which are instead expected to diffuse through the H-bond network of water molecules (i.e., Grotthuss-based mechanism [2]). In the early 2000s, the release of the first high-resolution AQP structures allowed to hypothesize that water molecules move in a single row through the channel and that the lack of a continuous hydrogen bond network prevents proton conduction via Grotthuss mechanism [6]”. The phrase “H95 might by responsible” should be replaced with “H95 might be responsible” in Section 3.3.1.

These changes do not affect the scientific results. The manuscript will be updated and the original will remain online on the article webpage.

## References

[1] Mangiatordi G.F., Alberga D., Trisciuzzi D., Lattanzi G., Nicolotti O. (2016). Human aquaporin-4 and molecular modeling: Historical perspective and view to the future. Int. J. Mol. Sci..

[2] Agmon N. (1995). The Grotthuss mechanism. Chem. Phys. Lett..

[3] Carbrey J.M., Agre P., Beitz P.D.E. (2009). Discovery of the aquaporins and development of the field. Handbook of Experimental Pharmacology.

[4] Verkman A.S., Mitra A.K. (2000). Structure and function of aquaporin water channels. Am. J. Physiol..

[5] Zeidel M.L., Ambudkar S.V., Smith B.L., Agre P. (1992). Reconstitution of functional water channels in liposomes containing purified red cell CHIP28 protein. Biochemistry.

[6] Sui H., Han B.G., Lee J.K., Walian P., Jap B.K. (2001). Structural basis of water-specific transport through the AQP1 water channel. Nature.

